# Biodistribution and radiation dosimetry of [^18^F]-JK-PSMA-7 as a novel prostate-specific membrane antigen-specific ligand for PET/CT imaging of prostate cancer

**DOI:** 10.1186/s13550-019-0540-7

**Published:** 2019-07-25

**Authors:** Melanie Hohberg, Carsten Kobe, Philipp Krapf, Philipp Täger, Jochen Hammes, Felix Dietlein, Boris D. Zlatopolskiy, Heike Endepols, Markus Wild, Stephan Neubauer, Axel Heidenreich, Bernd Neumaier, Alexander Drzezga, Markus Dietlein

**Affiliations:** 1 Department of Nuclear Medicine, University Hospital of Cologne, Kerpener Str. 62, 50937 Cologne, Germany; 2 Cancer Center Cologne, University Hospital of Cologne, Cologne, Germany; 3 Department of Medical Oncology, Dana-Farber Cancer Institute, Boston, MA 02215 USA; 4 Institute of Radiochemistry and Experimental Molecular Imaging, University Hospital of Cologne, Cologne, Germany; 5 Institute of Neuroscience and Medicine, INM-5 (Nuclear Chemistry), Research Center Juelich GmbH, Juelich, Germany; 6 West-German Prostate Center, Klinik am Ring, Cologne, Germany; 7 Department of Urology, University Hospital of Cologne, Cologne, Germany

**Keywords:** Dosimetry, PSMA, Prostate cancer, PET

## Abstract

**Aim:**

We investigated the whole-body distribution and the radiation dosimetry of [^18^F]-JK-PSMA-7, a novel ^18^F-labeled PSMA-ligand for PET/CT imaging of prostate cancer.

**Methods:**

Ten patients with prostate cancer and biochemical recurrence or radiologic evidence of metastatic diseases were examined with 329–384 MBq (mean 359 ± 17 MBq) [^18^F]-JK-PSMA-7. Eight sequential positron emission tomography (PET) scans were acquired from 20 min to 3 h after injection with IRB approval. The kidneys, liver, lungs, spleen, and salivary glands were segmented into volumes of interest using the QDOSE dosimetry software suite (ABX-CRO, Germany). Absorbed and effective dose were calculated using the ICRP-endorsed IDAC 1.0 package. The absorbed dose of the salivary glands was determined using the spherical model of OLINDA 1.1. PSMA-positive lesions were evaluated separately. Quantitative assessment of the uptake in suspicious lesions was performed by analysis of maximum (max) and peak SUV values. The gluteus maximus muscle (SUV_mean_) served as a reference region for the calculation of tumor-to-background ratios (TBR’s).

**Results:**

Physiologic radiotracer accumulation was observed in the salivary and lacrimal glands, liver, spleen, and intestines, in a pattern resembling the distribution known from other PSMA-tracers with excretion via urinary and biliary pathways. The effective dose from [^18^F]-JK-PSMA-7 for the whole body was calculated to be 1.09E−02 mGy/MBq. The highest radiation dose was observed in the kidneys (1.76E−01 mGy/MBq), followed by liver (7.61E−02 mGy/MBq), salivary glands (4.68E−02 mGy/MBq), spleen (1.89E−02 mGy/MBq), and lungs (1.10E-2 mGy/MBq). No adverse effects of tracer injection were observed. Six out of ten patients were scored as PSMA-positive. A total of 18 suspicious lesions were analyzed, which included six bone lesions, nine lymph nodes, and three local lesions within the prostate fossa. The values for the SUV_max_ and SUV_peak_ in the PSMA-positive lesions increased until 60 min p.i. and remained at this intensity in the PET/CT scans until 140 min. In the period between 170 and 200 min after injection, a further significant increase in SUV_max_ and SUV_peak_ within the PSMA-positive lesions was observed.

**Conclusions:**

The highest TBR of [^18^F]-JK-PSMA-7 was found 3 h after injection. From the kinetically collected data, it can be concluded that this trend may also continue in the further course. The start of the PET/CT acquisition should be chosen as late as possible. The high uptake in suspicious lesions in terms of absolute SUV_max_ and relative TBR values indicates potentially high sensitivity of the tracer for detection of prostate cancer manifestations.

## Introduction

The use of PET tracers targeting the prostate-specific membrane antigen (PSMA) for imaging in patients with prostate cancer is gaining increasing interest and has shown great promise for improving the treatment of patients with prostate cancer [1].

A decisive breakthrough was achieved with low-molecular ligands that bind to the active center of the extracellular domain of prostate-specific membrane antigen [2, 3]. It is therefore not surprising that great hopes are placed in improved diagnostics with PSMA-specific tracers for prostate cancer detection. In the meantime, a large number of small molecule PSMA ligands have been developed for use in PET imaging (e.g., [^68^Ga]-PSMA-11 [4], [^68^Ga]-PSMA I&T [5], [^18^F]-DCFBC [6], [^18^F]-DCFPyL [7–9], [^18^F]-PSMA-1007 [10]). Most of these tracers have a fast renal clearance, which enables prompt PET diagnosis with a good lesion-to-background contrast. [^18^F]-PSMA-1007 is characterized by a predominately hepatic-biliary excretion. Most clinical experience has been gained with [^68^Ga]-PSMA-11. However, ^68^Ga has several disadvantages with regard to decay properties and accessibility in comparison to ^18^F-labeled PSMA compounds and is of limited availability due to its production in radionuclide generators. Furthermore, compared to ^18^F (mean 0.65 MeV), the positron energy of ^68^Ga is higher (mean 1.90 MeV), reducing the theoretical maximum achievable spatial resolution [11].

The first generation of ^18^F-labeled PSMA-ligands, [^18^F]-DCFBC, was described by Mease et al. [12]. Images obtained with [^18^F]-DCFBC demonstrated a relatively high background activity [6]. This potential limitation has been addressed by the second generation of ^18^F-labeled PSMA-ligands, including [^18^F]-DCFPyL and [^18^F]-PSMA-1007. [^18^F]-PSMA-1007 offers the advantage that nuclides for diagnostics and therapy can be bound via a chelator [10]. For most of these PSMA-ligands, a complex manufacturing process is necessary [13–17]. The goal of this study was to investigate the whole-body distribution, radiation dosimetry, and safety of [^18^F]-JK-PSMA-7 in patients with known prostate cancer. [^18^F]-JK-PSMA-7 is a novel ^18^F-labeled PSMA-ligand with similar properties to other renally excreted ^18^F-labeled PSMA-ligands but with a “minimalistic light” manufacturing protocol as proposed by Richarz et al. [18] and by Neumaier et al. [19]. The abbreviation JK refers to the Research Center Jülich and University Hospital of Cologne involved. [^18^F]-JK-PSMA-7 was successfully tested in a preclinical setting on LNCaP C4-2 prostate tumor cells and on healthy Long Evans rats [20]. The most important results are briefly summarized here.

The uptake in LNCaP C4-2 cells of [^18^F]-JK-PSMA-7 was significantly higher than [^18^F]-DCFPyL after 2 h. The highest acutance was observed for [^18^F]-JK-PSMA-7 and [^18^F]-PSMA-1007 compared to [^18^F]-DCFPyL and [^68^Ga]-PSMA-11. Among all investigated tracers, [^18^F]-JK-PSMA-7 exhibited the highest resolution. Blood radioactivity of [^18^F]-JK-PSMA-7 was significantly lower compared to [^68^Ga]-PSMA-11 and [^18^F]-PSMA-1007 but in the same order of magnitude as [^18^F]-DCFPyL. The longer retention of blood radioactivity reflected in a higher background activity for [^68^Ga]-PSMA-11 and [^18^F]-PSMA-1007.

## Material and methods

### Patients

Between December 2017 and March 2018, 10 patients were subjected to [^18^F]-JK-PSMA-7 PET/CT due to the progression of prostate cancer. Eight patients suffered from biochemical recurrence (BCR). Two patients had metastasized castration-resistant prostate cancer (mCRPC) and received androgen deprivation therapy (ADT).

PET/CT imaging was performed in accordance with the Institutional Review Board. All patients gave written informed consent to PET imaging and inclusion of their data in a retrospective analysis. All procedures were performed in compliance with the regulations of the local authorities responsible (District Administration of Cologne, Germany).

### Radiosynthesis of [^18^F]-JK-PSMA-7

[^18^F]-JK-PSMA-7 was prepared in two steps according to the guidelines of a good manufacturing practice. First, 2,3,5,6-tetrafluorophenyl-6-([^18^F]fluoro)-4-methoxynicotinate ([^18^F]-Py-OMe-Tfp) was prepared by the reaction of [^18^F]fluoride with 2-methoxy-N,N,N-trimethyl-5-((2,3,5,6-tetrafluorophenoxy)carbonyl)pyridine-2-aminiumtrifluoro-methanesulfonate in a mixture of EtOH, MeCN, and tBuOH. The activated ester was purified by SPE purification and added to a freshly prepared ethanolic solution of (((S)-5-amino-1-carboxypentyl)carbamoyl)-l-glutamic acid (HO-Lys-C(O)-Glu-OH) and tetraethylammonium bicarbonate in EtOH. After coupling, the reaction mixture was purified by preparative HPLC. Radiochemical yield of [^18^F]-JK-PSMA-7 amounted to 30% (non-decay corrected). The obtained specific activity was 380 ± 16 GBq/μmol and the volumic activity 668 ± 38 MBq/ml. The [^18^F]-JK-PSMA-7 solution was administered to the patient by intravenous injection (mean 359.3 MBq, SD 17.1 MBq, range 329–384 MBq). More details about the synthesis and automated production of ^18^F-JK-PSMA-7 have recently been published [20].

### PET/CT acquisition and image reconstruction

All PET/CT scans were performed on a Siemens Biograph mCT (mCT 128 Flow Edge, Siemens, Knoxville, USA). In total, eight sequential whole-body PET scans were acquired from the base of the skull to mid-thigh. Patients were injected with 359 ± 17 MBq (4.25 ± 0.55 MBq/kg) of [^18^F]-JK-PSMA-7 by a slow intravenous push. The PET acquisition was subdivided into two blocks and the patients into two groups of five according to the following scheme: in patient group no. 1, the first acquisition block started at 20 min p.i. and was repeated four times every 10 min up to 50 min p.i. After a break of 60 min, the second acquisition block started at 110 min p.i and was again repeated four times every 10 min up to 140 min p.i. During an intermission, the patients were allowed to leave the table to void as needed. For each acquisition block, a low-dose non-enhanced CT (120 kV, mA modulation, pitch 1.2, slice thickness 5.0 mm) was performed for attenuation correction. A similar procedure was used in patient group no. 2, the only difference being that the first PET scan was acquired 80 min p.i. and the last one 200 min p.i. All emission data were corrected for attenuation, randoms, scatter, and decay. Reconstruction was conducted with an ordered subset expectation maximization (OSEM) algorithm with 4 iterations and 12 subsets and Gauss-filtered to a transaxial resolution of 5 mm at full-width at half-maximum (FWHM).

### Radiation dosimetry

Radiation dosimetry was performed using the QDOSE dosimetry software suite (ABX-CRO, Dresden, Germany). All PET and CT data sets were automatically co-registered. Non-target organs such as the kidneys, liver, spleen, lungs, and salivary gland were segmented into volumes of interest (VOI). Time activity curves (TAC) were calculated for the segmented organs. Curve fitting and integration was applied to all TACs to obtain the cumulated activity and residence time. The calculation of cumulated activity was divided into the following three sections: between time 0 and the first measuring point, a linear increase of the TAC was assumed. All measuring points were integrated numerically using trapezoidal approximation. From the last measuring point to infinity, a mono-exponential function was fitted over the last four measuring points and integrated. Whereas the absorbed dose to the salivary glands was determined using the spherical model from OLINDA/EXM1.1 [21], the effective dose and absorbed partial and whole-body dose were calculated using the ICRP endorsed IDAC 1.0 package [22].

### Biodistribution

The biodistribution of [^18^F]-JK-PSMA-7 was quantified by maximum (max) and mean SUV values for all sequentially acquired PET images. The standardized uptake value (SUV) was defined by a circular region drawn around an area with focally increased uptake and automatically adapted to a three-dimensional VOI by software (syngo.via VB20A, Siemens, Erlangen, Germany). The kidneys, liver, spleen, lungs and salivary glands were evaluated with a VOI of 2–3 cm in diameter placed inside the organ parenchyma. PSMA-positive lesions were analyzed separately.

Blood radioactivity concentration was measured in the lumen of the left ventricle of the heart for all sequentially acquired PET images. Quantification was performed for the total blood volume, which was estimated by size, weight, and hematocrit [23]. For this purpose, mean values of the blood radioactivity concentration from both patient groups were determined.

### PSMA-positive lesions

All PET/CT images were reviewed and analyzed on a syngo.via workstation (syngo.via VB20A Software, Siemens, Erlangen, Germany) by two experienced nuclear medicine physicians (both with 20 years’ experience in PET scan reading, certificate for CT reading). The visual assessment of [^18^F]-JK-PSMA-7 was considered to be positive if there was focal uptake above the mediastinal blood pool or the liver value. Spherical volumes of interest (VOIs) were manually drawn around areas with focally increased uptake. Quantitative assessment of the uptake in lesions was performed by analysis of maximum (max) and peak SUV values. SUV_max_ is defined as the hottest voxel within a volume of interest. SUV_peak_ computes the mean SUV within a 1-cm^3^ sphere positioned within a VOI so as to maximize that mean. In doing so, voxel super-sampling is performed where the dimensions are halved until they are less than or equal to 0.5 mm on each axis [24]. Tumor-to-background ratios (TBRs) were calculated based on the following equation [25]:

1 $$ \mathrm{TBR}=\frac{{\mathrm{SUV}}_{\mathrm{max}}\left(\mathrm{lesion}\right)}{{\mathrm{SUV}}_{\mathrm{mean}}\left(\mathrm{reference}\right)} $$

### Statistical analysis

The software package SPSS Statistics 25 (IBM, Armonk, USA) was used for statistical analysis. Dosimetric differences between [^18^F]-JK-PSMA-7 and other common ^18^F-labeled PSMA-ligands ([^18^F]-DCFBC [6], [^18^F]-DCFPyL [7], [^18^F]-PSMA-1007 [10]) were compared by a Mann-Whitney test. A *p* value of < 0.05 was considered statistically significant.

## Results

### Patient characteristics

The patients were on average 69 years old (range 52–76 years), with an average weight of 86 kg (range 74–100 kg). The average PSA value was 15.6 μg/L (range 0.51–130 μg/L). Nine patients had undergone radical prostatectomy, and one patient had external beam radiation therapy for primary treatment. Two patients received androgen deprivation therapy (ADT) within the last 6 months prior to the examination. Detailed patient characteristics are summarized in Table 1.

**Table 1:** Patient characteristics

Patient, no.	Age [years]	PSA [μg/L]	PSA doubling time [months]	Indication Gleason	Activity [MBq]	Activity/weight [MBq/kg]	Local PSMA+	Nodal PSMA+	Distant PSMA+	Therapeutic consequence
1	76	1.48 ADT	8	RT, ADT. Intensification of the ADT? 4 + 4	363	4.78	Right prostate	0	Sternum	RT prostate. No intensification of the ADT
2	67	0.7	9	BCR after prostatectomy 4 + 3	355	4.03	0	2 LNs left iliacal	0	RT LNs
3	66	1.03	> 12	BCR after prostatectomy and RT 4 + 3	347	3.62	0	0	0	Wait and see
4	74	1.1	4	BCR after prostatectomy 3 + 4	350	4.73	0	1 LN left iliacal	0	S-LAD and RT LN
5	63	4.7	n.a.	BCR after prostatectomy. 4 + 3	329	3.78	Right prostate	0	0	RT prostate fossa
6	64	130.0 ADT	3	Prostatectomy, ADT. Planning local intervention? n.a.	384	3.96	Right prostate	2 LNs pre-sacral 1 LN right iliacal	> 5 osseous lesions	No cystectomy
7	52	14.9	> 12	BCR after prostatectomy and RT 3 + 3	370	4.30	0	0	0	Wait and see
8	73	0.8	5	BCR after prostatectomy 4 + 3	371	4.95	0	3 LNs retroperitoneal	0	RT LNs
9	74	1.02	> 12	BCR after prostatectomy 3 + 4	379	4.6	0	0	0	Wait and see
10	59	0.51	n.a.	BCR after prostatectomy 4 + 3	345	3.45	0	0	0	RT prostate fossa

*ADT* androgen deprivation therapy, *BCR* biochemical recurrence, *LAD* lymphadenectomy, *LN* lymph node, *n.a.* not available, *RT* radiotherapy, *S-LAD* salvage lymphadenectomy

### Biodistribution

**Fig. 1:**
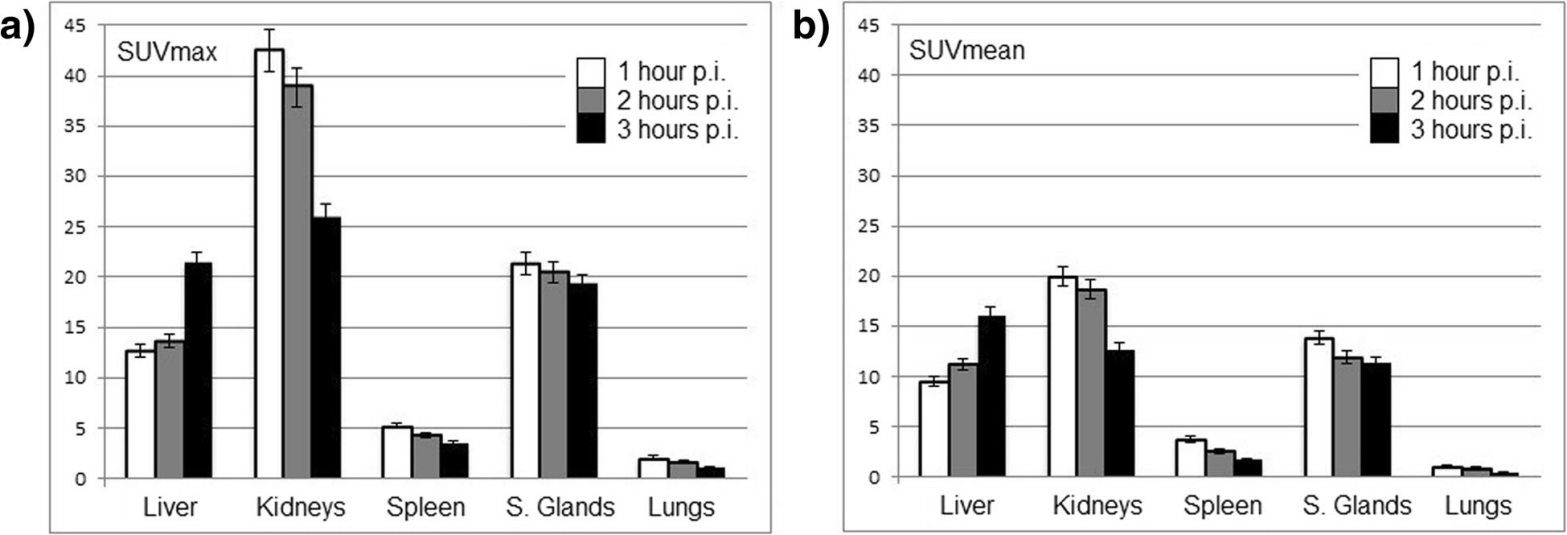
Biodistribution of **a** SUV_max_ and **b** SUV_mean_ of [18F]-JK-PSMA-7 in non-target organs over time with standard deviations

Physiologic radiotracer accumulation was observed in the salivary and lacrimal glands, liver, spleen, and intestines, in a pattern resembling the distribution known from other PSMA tracers with excretion via urinary and biliary pathways. SUV_max_ and SUV_mean_ as well as their change over time in standard organs are presented in Fig. 1. Between 1 h and 3 h p.i., the uptake in the liver demonstrated an increase in SUV_max_ and SUV_mean_ of around 69%, whereas the uptake in the other standard organs showed a decrease in SUV_max_ and SUV_mean_, amounting to − 37% for the kidneys, − 41% for the spleen, − 45% for the lungs, and − 14% for the salivary glands (mean values).

**Fig. 2:**
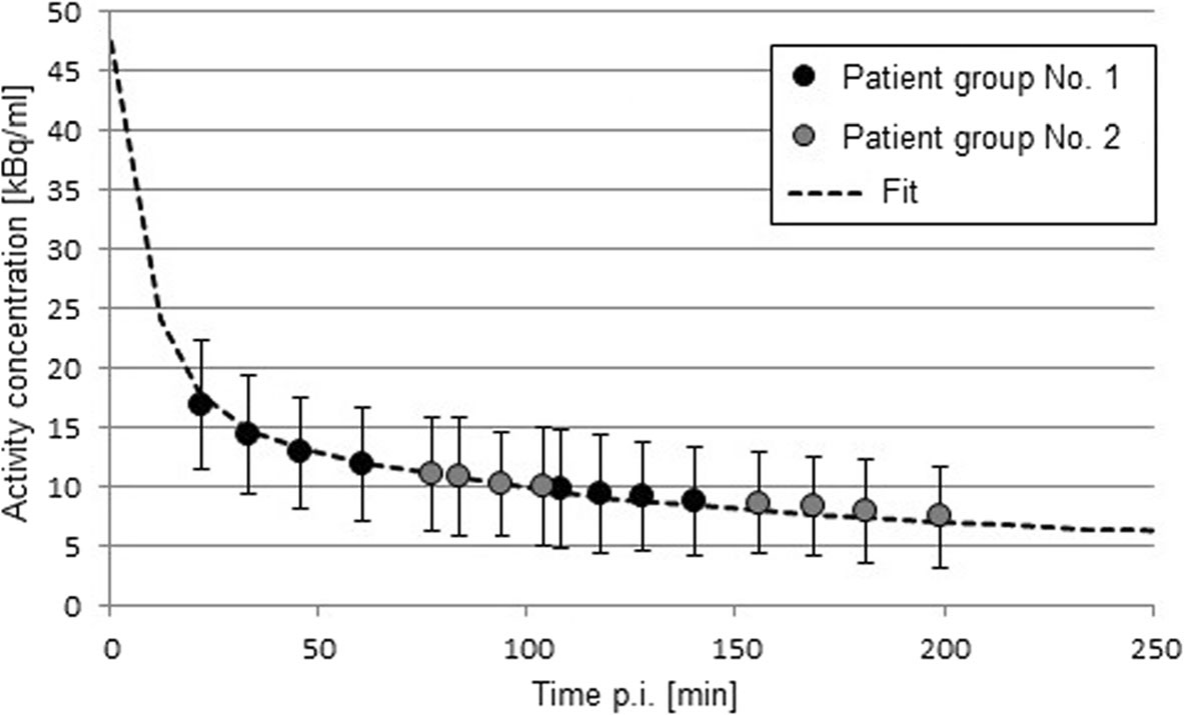
Average blood activity concentration of [^18^F]-JK-PSMA-7 derived from both patients groups. All error bars refer to the standard deviation

As can be seen in Fig. 2, fast excretion via the blood is evident. From the exponential fit of the TAC, an average half-life of the blood activity concentration of 74 min can be calculated. The whole blood pool contained in mean 13%, 8%, and 4% of the injected activity at 1 h, 2 h, and 3 h p.i., respectively. These initial clinical data are in good agreement with the preclinical data collected in rats [20].

### Radiation dosimetry

The effective dose from [^18^F]-JK-PSMA-7 for the whole body was calculated to be 1.09E−02 mGy/MBq. The highest radiation dose was observed in the kidneys (mean 1.76E−01 mGy/MBq), followed by liver (mean 7.61E−02 mGy/MBq), salivary glands (mean 4.75E−02 mGy/MBq), spleen (mean 1.89E−02 mGy/MBq), and lungs (1.10E-2 mGy/MBq). The individual results for each patient’s whole body and partial organ absorbed doses are presented in Table 2. For comparison, Table 3 presents the corresponding absorbed doses and residence times for other ^18^F-labeled PSMA-ligands (PSMA-1007, DCFPyL, DCFBC). As in this work, the QDOSE dosimetry software suite was used for [^18^F]-PSMA-1007. Other software packages were used for [^18^F]-DCFBC and [^18^F]-DCFPyL, but radiation dosimetry was performed in a similar way.

**Table 2:** Absorbed dose [mGy/MBq] for all segmented organs for each patient

Organ	Patient no. 1	Patient no. 2	Patient no. 3	Patient no. 4	Patient no. 5	Patient no. 6	Patient no. 7	Patient no. 8	Patient no. 9	Patient no. 10
Kidneys	1.44E−02	2.66E−01	1.74E−01	2.15E−01	1.34E−01	1.29E−01	2.90E−01	7.45E−02	1.72E−01	1.58E−01
Lungs	9.84E−03	1.07E−02	1.24E−02	1.46E−02	9.92E−03	1.19E−02	6.61E−03	9.95E−03	1.19E−02	1.26E−02
Liver	6.40E−02	9.22E−02	8.30E−02	4.22E−02	7.25E−02	9.94E−02	5.95E−02	7.68E−02	7.13E−02	1.00E−01
Spleen	3.04E−02	1.67E−02	1.86E−02	6.92E−03	1.08E−02	1.31E−02	2.12E−02	1.47E−02	2.38E−02	3.27E−02
Salivary glands	6.57E−02	4.13E−02	7.97E−02	4.87E−02	3.12E−02	4.16E−02	3.53E−02	4.82E−02	3.84E−02	3.74E−02
Effective dose	8.64E−03	1.46E−02	1.19E−02	1.08E−02	8.85E−03	1.16E−02	1.30E−02	6.61E−03	1.02E−02	1.26E−02

**Table 3:** Dosimetry comparison of [18F]-JK-PSMA-7 with other ^18^F-labled PSMA tracers

Organ	[^18^F]-JK-PSMA-7 This work	[^18^F]-PSMA-1007 Giesel et al. [10]	[^18^F]-DCFPyL Szabo et al. [7]	[^18^F]-DCFBC Cho et al. [6]
Mean absorbed dose [mGy/MBq]				
Number of patients	10	3	4	5
Used dosimetry software	QDOSE, ABX-CRO, Germany	QDOSE, ABX-CRO, Germany	MIM Software, Cleveland, Ohio	ANALYZE, BIR, Mayo Clinic
Kidneys	1.76E−01 (6.52E−02 SD)	1.70E−01 (3.09E−02 SD)	9.45E−02 (n.a.)	2.84E−02 (3.81-03 SD)
Lungs	1.10E−02 (2.16E−03 SD)	1.11E−02 (2.60E−04 SD)	1.08E−02 (n.a.)	2.45E−02 (2.99-03 SD)
Liver	7.61E−02 (1.83E−02 SD)	6.02E−02 (6.24E−04 SD)	3.80E−02 (n.a.)	2.46E−02 (4.16-03 SD)
Spleen	1.89E−02 (8.28E−03 SD)	7.39E−02 (2.96E−02 SD)	1.85E−02 (n.a.)	1.72E−02 (1.05E−03 SD)
Salivary glands	4.68E−02 (1.50E−02 SD)	9.00E−02 (n.a.)	2.68E−02 (n.a.)	n.a.
Effective dose	1.09E−02 (2.37E−03 SD)	2.20E−02 (2.08E−04 SD)	1.39E−02 (n.a.)	1.99E−02 (1.34E−03 SD)
Mean residence time [MBq·h/MBq]				
Kidneys	2.26E−01 (7.83E−02 SD)	2.64E−01 (5.03E−03 SD)	2.17E−01 (n.a.)	3.50E−02 (5.84E−03 SD)
Lungs	4.39E−02 (7.16E−03 SD)	n.a.	3.70E−02 (n.a.)	1.09E–01 (1.87E−02 SD)
Liver	5.70E−01 (1.31E −01 SD)	4.42E−01 (2.87E−02 SD)	2.60E−01 (n.a.)	1.59E−01 (3.29E−02 SD)
Spleen	2.32E−02 (7.09E−03)	6.29E−02 (2.89E−02 SD)	2.07E−02 (n.a.)	1.01E−02 (8.92E−04 SD)
Salivary glands	1.75E−02 (2.68E−03 SD)	1.37E−02 (3.64E−03 SD)	6.95E−03 (n.a.)	n.a.
Whole-body	1.93E+00 (1.48E−01 SD)	2.64E+00 (0.00 SD)*	1.96E+00 (n.a.)	2.37E+00 (9.09E−02 SD)

*n.a.* not available, *SD* standard deviation

*The residence time for the whole-body was the same for all three patients [10]

### Statistical comparison with other PSMA ligands

There was no statistically significant difference between the residence times of [^18^F]-JK-PSMA-7 and [^18^F]-PSMA-1007 for the examined organs (*p* > 0.05 for all organs). The comparison between [^18^F]-JK-PSMA-7 and [18F]-DCFPyL showed a statistically significant increase for residence times in the liver (47%, *p* = 0.034) and the salivary glands (40%, *p* = 0.003) for [^18^F]-JK-PSMA-7. For the kidneys (*p* > 0.05) and the spleen (*p* > 0.05), on the other hand, no significant difference in residence times was found. In the comparison between [^18^F]-JK-PSMA-7 and [^18^F]-DCFBC, a statistically significant increase was observed for the residence times in the kidneys (17%, *p* = 0.001) and the liver (28%, *p* = 0.002) for [^18^F]-JK-PSMA-7. There was no statistically significant difference for the residence time in the spleen and the lungs (*p* > 0.05).

### Visual analysis of PSMA-positive lesions

Six out of ten patients were scored as PSMA-positive. Readings were based on the interpretation of the latest PET-scans, performed 140 min and 200 min, respectively. A total of 18 suspicious lesions were analyzed, which included six bone lesions, nine lymph nodes, and three local lesions within the prostate fossa. Three of these six patients (patients no. 1, 2, and 4) belonged to patient group no. 1 (acquisition protocol 20 min–140 min p.i.) and the other three (patients no. 5, 6, and 8) to patient group no. 2 (acquisition protocol 80 min–200 min p.i.).

### Quantitative analysis of PSMA-positive lesions

**Fig. 3:**
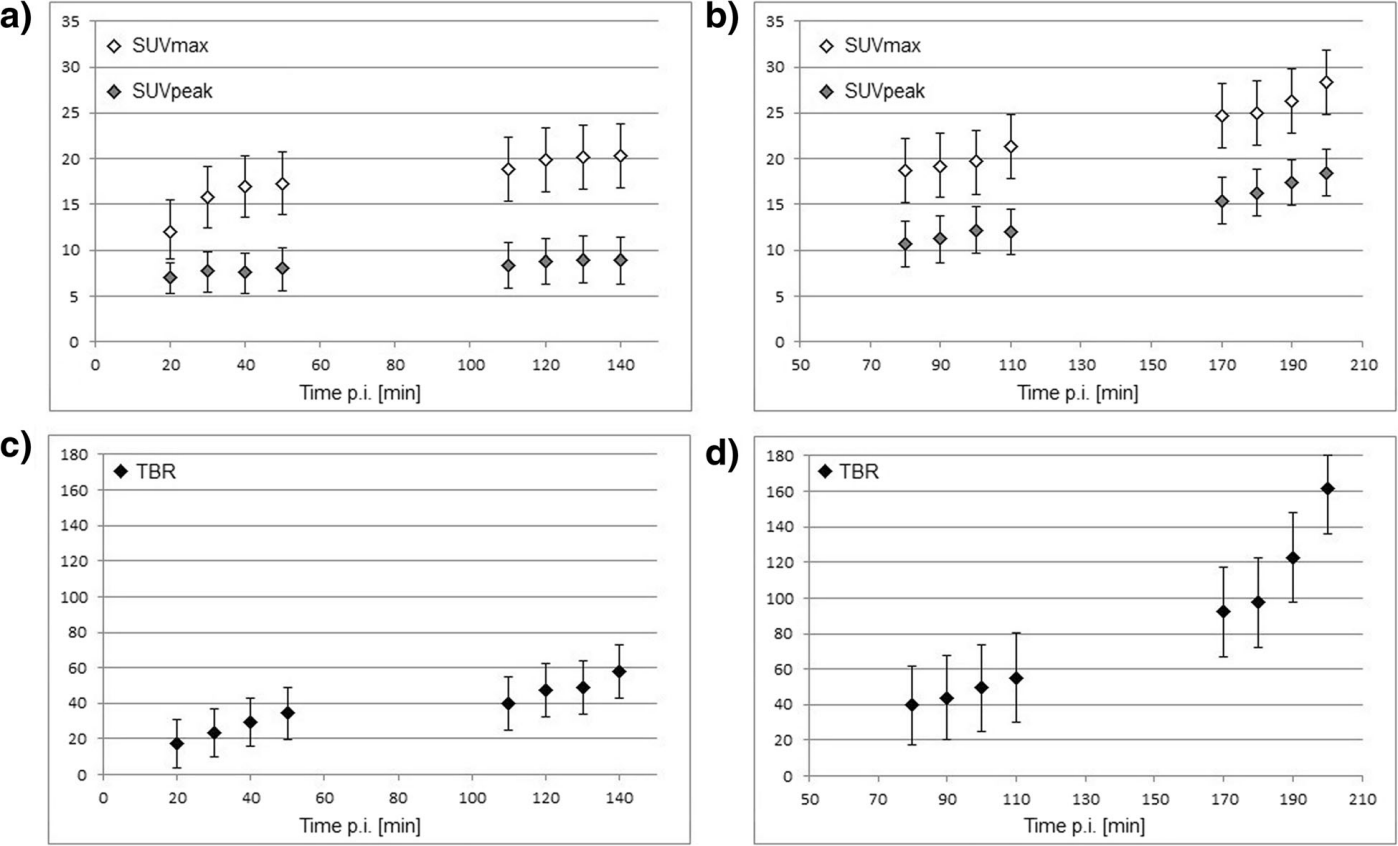
SUV_max_, SUV_peak_, and TBR of [^18^F]-JK-PSMA-7 in six patients with 18 PSMA-positive lesions. Time course of SUV_max_ and SUV_peak_ for patient group no. 1 (data acquisition between 20 and 140 min p.i., three patients, 6 lesions) exhibited in **a** and patient group no. 2 (data acquisition between 80 and 200 min p.i., three patients, 12 lesions) in **b**. Corresponding TBR for patient group no. 1 shown in **c** and patient group no. 2 in **d**. All error bars refer to the standard deviation

**Fig. 4:**
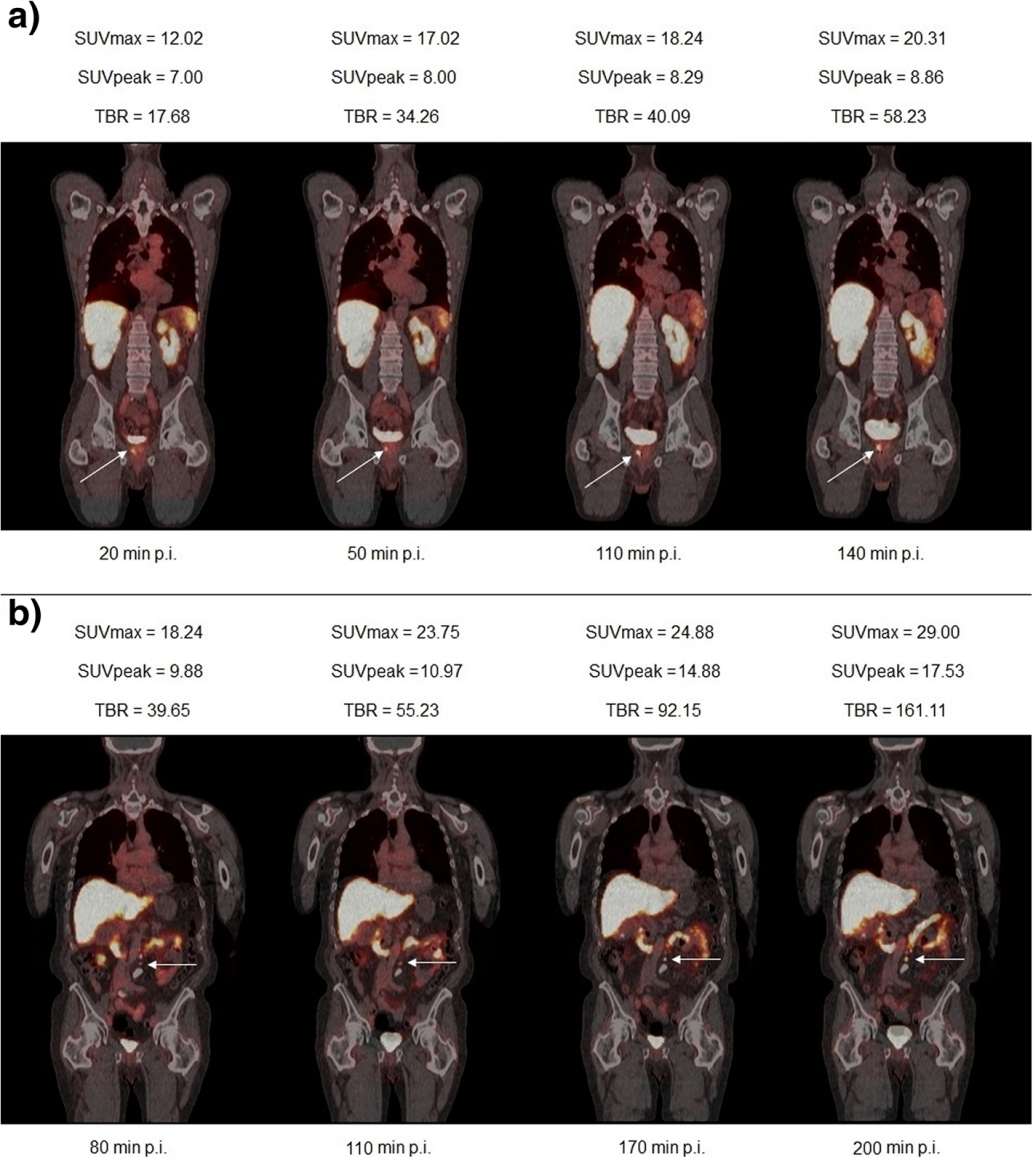
**a** [^18^F]-JK-PSMA-7 PET/CT scans obtained at 20 min, 50 min, 110 min, and 140 min p.i. and corresponding values for the SUV_max_, SUV_peak_, and TBR of one suspicious lesion within the prostate fossa (arrow). **b** [^18^F]-JK-PSMA-7 PET/CT scans obtained at 80 min, 110 min, 170 min, and 200 min p.i. and corresponding values for the SUV_max_, SUV_peak_, and TBR of a suspicious retroperitoneal lymph node (arrow). Here, we have quantified the middle lymph node from a PSMA-positive lymph node chain

All PSMA-positive lesions showed an increased uptake over time, although significant differences emerged between the two patient groups. In patient group no. 1, the SUV_max_ and SUV_peak_ values increase slowly over time and reach an almost stable plateau 100 min after injection. In patient group no. 2, on the other hand, the SUV_max_ and SUV_peak_ values continue to rise significantly. These differences in the time courses of SUV_max_ and SUV_peak_ are reflected in corresponding values for the TBR. Thus, between 100 and 140 min p.i., the mean TBR increased by 50%, whereas between 140 and 200 min p.i., the mean TBR increased by 78%. Figure 3 shows the time course of the SUV_max_, SUV_peak_, and TBR in PSMA-positive lesions. In addition, Fig. 4 shows the images of two patients, one from patient group no. 1 and one from patient group no 2.

### Adverse events

All patients tolerated the examination well. No drug-related pharmacological effects or physiologic responses were observed. None of the patients reported any adverse events or side-effects at the time, when the PET-results were discussed with the patient or some weeks later, when the therapeutic consequences were discussed by telephone counseling.

### Verification

Five out of the 6 patients with PSMA-positive lesions showed a decreased PSA level after radiotherapy of the PSMA-positive lymph nodes or after salvage radiotherapy of the PSMA-positive lesions within the prostate fossa. One patient had multiple PSMA-positive lymph nodes and skeletal lesions, which were also visible on the CT scan.

Two out of the 4 patients with a PSMA-negative PET result had a stable PSA level after 6 to 8 months without any therapy. One patient received salvage radiotherapy and the PSA level decreased. One patient developed osteosclerotic bone metastases after 9 months, then visible on the CT scan, but again PSMA-negative with [^68^Ga]-PSMA-11.

## Discussion

Ten patients were examined with [^18^F]-JK-PSMA-7. In summary, the observation of patients with the novel tracer can be summarized as follows.

The mean effective dose was comparable to that of other ^18^F-labeled PSMA ligands. Furthermore, the preserved organ doses were within the same order of magnitude as other ^18^F-labeled PSMA ligands. One problem with the comparison of organ doses is that in previous studies, the organ masses have been determined differently. In some cases, the masses were determined individually for each patient using CT images [10] while elsewhere reference organ masses according to ICRP were used [7]. A comparison of the different tracers on the basis of the residence times and thus independent of mass is therefore much more conclusive. Regarding the tracer residence times in various organ systems, no significant difference was found between [^18^F]-JK-PSMA-7 and [^18^F]-PSMA-1007. Significant increases were found for the liver in comparison to DCFBC (28%, *p* = 0.002) and DCFPyL (47%, *p* = 0.034). It should be noted here that the mean values from the other studies are based on very small cohorts (usually only three to five patients). In our study, the mean values are based on ten examined patients.

[^18^F]-JK-PSMA-7 showed a fast excretion via the blood in a similar order of magnitude to [^18^F]-DCFPyL. Zlatopolskiy et al. [20] were able to show that the blood protein binding of [^18^F]-PSMA-1007 and [^68^Ga]-PSMA-11 was significantly higher compared to [^18^F]-JK-PSMA-7. High blood protein binding may delay the excretion of the tracer resulting in lower accumulation in kidneys and bladder. This could be advantageous for detecting PCa metastases adjacent to urethra and bladder, while a faster excretion would favorably affect the background enrichment.

A major strength of this study is that we investigated the temporal course of SUV values not only for organs but also for ^18^F-PSMA-positive lesions over a long period of time. The values for SUV_max_ and SUV_peak_ in the PSMA-positive lesions increased for up to 60 min p.i. and remained at this intensity in the subsequent PET/CT scans up to 140 min p.i. In the period between 170 and 200 min after injection, further significant increases in SUV_max_ and SUV_peak_ were seen in PSMA-positive lesions. From this data, it can be concluded that a late acquisition window, even up to 3 h after injection, may be favorable for this tracer. This also confirms the observations made from evaluation of Lu^177^-PSMA therapies. The tracer uptake in PSMA-positive lesions was found to increase up to 24 h after injection, in contrast to uptake in non-target organs, which mainly peaked on the day of treatment, and declined thereafter [26]. Therefore, there might be a clear advantage compared to [^68^Ga]-PSMA-11. The direct comparison of [18F]-JK-PSMA-7 with [^68^Ga]-PSMA-11 and other ^18^F-labeled PSMA-ligands will be the subject of another work by our department [27].

One limitation of this study is that [^18^F]-JK-PSMA-7 was not tested on healthy volunteers. But there is no reason to assume that normal organs, when free of lesions, have a different tracer uptake in healthy men compared to patients. For [^18^F]-DCFPyL, the dosimetry in normal organs was proven to be assessed independently of tumor presence [7].

## Conclusion

The radiation dose from [^18^F]-JK-PSMA-7 was similar to that delivered by other ^18^F-labeled PSMA-ligands. The highest TBR was found 3 h after injection. From the kinetically collected data, it can be concluded that this trend may also continue in the further course. The start for the PET/CT acquisition should be chosen as late as possible. The high uptake in suspicious lesions in terms of absolute SUV_max_ and relative TBR values indicates potentially high sensitivity of the tracer for the detection of prostate cancer manifestations. In order to evaluate the potential sensitivity and optimized pharmacokinetics of [^18^F]-JK-PSMA-7, a larger prospective and comparative study is ongoing.

## Data Availability

The data generated during this simulation study are included in this published article or can be made available upon reasonable request.

## Abbreviations

ADT: Androgen deprivation therapy
BCR: Biochemical recurrence
CT: Computed tomography
[^18^F]-DCFBC: N-[N-[(S)-1,3-dicarboxypropyl]carbamoyl]-4-(18)F-fluorobenzyl-L-cysteine
[^18^F]-DCFPyL: 2-(3-{1-carboxy-5-[(6-[(18)F]fluoro-pyridine-3-carbonyl)-amino]-pentyl}-ureido)-pentanedioic acid
FWHM: Full-width at half-maximum
[^68^Ga]-PSMA-11: Glu-urea-Lys(Ahx)-HBED-CC
ICRP: International Commission on Radiological Protection
LNCaP C4-2: Prostate cancer cell line derived from the left supraclavicular lymph node metastasis
mCRPC: Metastasized castration-resistant prostate cancer
PET: Positron emission tomography
PSA: Prostate-specific antigen
PSMA: Prostate-specific membrane antigen
SUV: Standardized uptake value
TAC: Time activity curve
TBT: Tumor-to-background ratio
VOI: Volume of interest

## References

[1] Afshar-Oromieh A, Haberkorn U, Eder M, Eisenhut M, Zechmann CM (2012). [^68^Ga]gallium-labeled PSMA ligands as superior PET tracer for the diagnosis of prostate cancer: comparison with ^18^F-FECH. Eur J Nucl Med Mol Imaging..

[2] Luthi-Carter R, Barczak AK, Speno H, Coyle JT (1998). Molecular characterization of human brain N-acetylated α-linked acidic dipeptidase (NAALADase). J Pharmacol Exp Ther..

[3] Tiffany CW, Lapidus RG, Merion A, Calvin DC, Slusher BS (1999). Characterization of the enzymatic activity of PSM: comparison with brain NAALADase. Prostate..

[4] Eder M, Schäfer M, Bauder-Wüst U, Hull WE, Wängler C, Mier W (2012). ^68^Ga-complex lipophilicity and the targeting property of a urea-based PSMA inhibitor for PET imaging. Bioconjug Chem..

[5] Herrmann K, Blümel C, Weineisen M, Schottelius M, Wester HJ, Czernin J (2015). Biodistribution and radiation dosimetry for a novel probe targeting prostate specific membrane antigen for Imaging and Therapy (^68^Ga-PSMA I&T). J Nucl Med.

[6] Cho SY, Gage KL, Mease RC, Senthamizhchel Van S, Holt DP, Jeffrey-Kwanisai (2012). Biodistribution, tumor detection, and radiation dosimetry of 18F-DCFBC, a low-molecular-weight inhibitor of prostate specific membrane antigen, in patients with metastatic prostate cancer. J Nucl Med..

[7] Szabo Z, Mena E, Rowe SP, Plyku D, Nidal R, Eisenberger MA (2015). Initial evaluation of [^18^F]DCFPyL for prostate-specific membrane antigen (PSMA)-targeted PET imaging of prostate cancer. Mol Imaging Biol..

[8] Dietlein M, Kobe C, Kuhnert G, Stockter S, Fischer T, Schomäcker K (2015). Comparison of [18F]DCFPyl and [68Ga]Ga-PSMA-HBED-CC for PSMA-PET imaging in patients with relapsed prostate cancer. Mol Imaging Biol..

[9] Dietlein F, Kobe C, Neubauer S, Schmidt M, Stockter S, Fischer T (2017). PSA-stratified performance of ^18^F- and ^68^Ga-PSMA PET in patients with biochemical relapse of prostate cancer. J Nucl Med..

[10] Giesel FL, Hadaschik B, Cardinale J, Radtke J (2017). F-18 labeled PSMA-1007: biodistribution, radiation dosimetry and histopathological validation of tumor lesions in prostate cancer patients. Eur J Nucl Med Mol Imaging..

[11] Sanchez-Crespo A (2013). Comparison of Gallium-68 and Fluorine-18 imaging characteristics in positron emission tomography. Appl Radiat Isot..

[12] Mease RC, Dusich CL, Foss CA, Ravert HT, Dannals RF, Seidel J (2008). Synthesis and *in vivo* evaluation of N-[N-[[S]-1 ,3-Dicarboxypropyl]carbamoyl]-4-[^18^F]fluorobenzyl-L-cysteine, [^18^F]DCFBC: a new imaging probe for prostate cancer. Clin Cancer Res..

[13] Benešová M, Bauder-Wüst U, Schäfer M, Klika KD, Mier W, Haberkorn U (2015). Linker modification strategies to control the prostate-specific membrane antigen (PSMA)-targeting and pharmacokinetic properties of DOTA-conjugated PSMA inhibitors. J Med Chem..

[14] Olberg DE, Arukwe JM, Grace D, Hjelstuen OK, Solbakken M, Kindberg GM (2010). One step radiosynthesis of 6-[18F]fluoronicotinic acid 2,3,5,6-tetraflourophenyl ester ([18F]-F-Py-TFP): a new prosthetic group for the efficient labeling of biomolecules with fluorine-18. J Med Chem.

[15] Cardinale J, Schäfer M, Benešová M, Bauder-Wüst U, Leotta K, Eder M (2017). Preclinical evaluation of 18F-PSMA-1007: a new PSMA-ligand for prostate cancer imaging. J Nucl Med..

[16] Chen Y, Pullambhatla M, Byun Y, Nimmaquadda S, Senthamizhchelvan S, Squouros G (2011). 2-(3-{1-Carboxy-5-[(6-[^18^F]fluoro-pyridine-3-carbonyl)-amino]-pentyl}-urei do)-pentanedioic acid, [^18^F]DCFPyL, a PSMA-based PET imaging agent for prostate cancer. Clin Cancer Res.

[17] Maresca KP, Hillier SM, Femia FJ, Keith D, Barone C, Joyal JL (2009). A series of halogenated heterodimeric inhibitors of prostate specific membrane antigen (PSMA) as radiolabeled probes for targeting prostate cancer. J Med Chem..

[18] Richarz R, Krapf P, Zarrad F, Urusova EA, Neumaier B, Zlatopolskiy BD (2014). Neither azeotropic drying, nor base nor other additives: a minimalist approach to 18F-labeling. Org Biomol Chem..

[19] Neumaier B, Zlatopolskiy BD, Richarz R, Krapf P. Method for the production of 18F-labeled active esters and their application exemplified by the preparation of a PSMA-specific PET-tracer. Patent WO2016030329A1. 2014.

[20] Zlatopolskiy Boris D., Endepols Heike, Krapf Philipp, Guliyev Mehrab, Urusova Elizaveta A., Richarz Raphael, Hohberg Melanie, Dietlein Markus, Drzezga Alexander, Neumaier Bernd (2018). Discovery of 18F-JK-PSMA-7, a PET Probe for the Detection of Small PSMA-Positive Lesions. Journal of Nuclear Medicine.

[21] Stabin MG, Sparks RB, Crowe E, OLINDA/EXM (2005). The second-generation personal computer software for internal dose assessment in nuclear medicine. J Nucl Med..

[22] Andersson M, Johansson L, Minarik D, Mattsson S, Leide-Svegborn S (2013). An internal radiation dosimetry computer program, IDAC2.0, for estimation of patient dose for radiopharmaceuticals. Radiat Prot Dosimetry..

[23] Gombotz H, Zacharowski K, Spahn DR. Patient Blood Management. Thieme. 2013; pp 79-80; ISBN: 9783132410770.

[24] syngo.via Operator Manual – syngo.MM oncology VB30. Print No. P02-005.621.01.01.02; Published by Siemens Healthcare GmbH.

[25] Delker A, Fendler WP, Kratochwil C, Brunegraf A, Gosewich A, Gildehaus FJ (2016). Dosimetry for ^177^Lu-DKFZ-PSMA-617: a new radiopharmaceutical for the treatment of metastatic prostate cancer. Eur J Nucl Med Mol Imaging..

[26] Dietlein M, Hohberg M, Kobe C, Dietlein F, Zlatopolskiy B, Krapf P (2018). Performance of the novel ^18^F-labeled prostate-specific membrane antigen-ligand PSMA-7 for PET/CT in prostate cancer patients. [abstract]. J Nucl Med..

